# Enhanced Auditory Brainstem Response and Parental Bonding Style in Children with Gastrointestinal Symptoms

**DOI:** 10.1371/journal.pone.0032913

**Published:** 2012-03-21

**Authors:** Shizuka Seino, Satoshi Watanabe, Namiko Ito, Konosuke Sasaki, Kaori Shoji, Shoko Miura, Kanoko Kozawa, Kunihiko Nakai, Hiroshi Sato, Motoyori Kanazawa, Shin Fukudo

**Affiliations:** 1 Department of Behavioral Medicine, Tohoku University Graduate School of Medicine, Sendai, Japan; 2 Health Science, Tohoku University Graduate School of Medicine, Sendai, Japan; 3 Department of Environmental Health Science, Tohoku University Graduate School of Medicine, Sendai, Japan; 4 Department of Clinical Physiology, Tohoku University Hospital, Sendai, Japan; Bremen Institute of Preventive Research and Social Medicine, Germany

## Abstract

**Background:**

The electrophysiological properties of the brain and influence of parental bonding in childhood irritable bowel syndrome (IBS) are unclear. We hypothesized that children with chronic gastrointestinal (GI) symptoms like IBS may show exaggerated brainstem auditory evoked potential (BAEP) responses and receive more inadequate parental bonding.

**Methodology/Principal Findings:**

Children aged seven and their mothers (141 pairs) participated. BAEP was measured by summation of 1,000 waves of the electroencephalogram triggered by 75 dB click sounds. The mothers completed their Children's Somatization Inventory (CSI) and Parental Bonding Instrument (PBI). CSI results revealed 66 (42%) children without GI symptoms (controls) and 75 (58%) children with one or more GI symptoms (GI group). The III wave in the GI group (median 4.10 interquartile range [3.95–4.24] ms right, 4.04 [3.90–4.18] ms left) had a significantly shorter peak latency than controls (4.18 [4.06–4.34] ms right, *p* = 0.032, 4.13 [4.02–4.24] ms left, *p* = 0.018). The female GI group showed a significantly shorter peak latency of the III wave (4.00 [3.90–4.18] ms) than controls (4.18 [3.97–4.31] ms, *p* = 0.034) in the right side. BAEP in the male GI group did not significantly differ from that in controls. GI scores showed a significant correlation with the peak latency of the III wave in the left side (rho = −0.192, p = 0.025). The maternal care PBI scores in the GI group (29 [26]–[33]) were significantly lower than controls (31 [28.5–33], *p* = 0.010), while the maternal over-protection PBI scores were significantly higher in the GI group (16 [12]–[17]) than controls (13 [10.5–16], *p* = 0.024). Multiple regression analysis in females also supported these findings.

**Conclusions:**

It is suggested that children with chronic GI symptoms have exaggerated brainstem responses to environmental stimuli and inadequate parental behaviors aggravate these symptoms.

## Introduction

Irritable bowel syndrome (IBS) is the most common functional gastrointestinal (GI) disorder (FGID) [1]. IBS is characterized by altered bowel habits and is associated with chronic abdominal pain and discomfort [2]. On the other hand, recurrent abdominal pain (RAP) during childhood is one of the most common pediatric disorders [3], [4], with epidemiological studies suggesting that 7–25% of school-age children suffer from RAP [5], [6]. RAP in childhood has been known to have visceral hypersensitivity sharing the pathophysiological features of childhood IBS [7] and to develop into adult IBS [8]. The prevalence of RAP increases with age into adolescence [8]. Age and gender have been shown to influence the prevalence of RAP, with an equal gender ratio in early childhood, with symptoms reported by girls predominately by late childhood [6], [9]. However, the pathogenesis and pathophysiology of IBS/RAP in childhood is complex and incompletely understood. It is not clear whether there is sex difference of pathophysiology of childhood IBS/RAP and the process of development into IBS.

In IBS patients, visceral hypersensitivity is one of the representative pathophysiology phenomena [10], [11]. Several studies using rectal barostats have confirmed the presence of visceral hypersensitivity by showing lower pain thresholds in children with IBS [4], [7], [12]. This enhanced sensitivity may underlie the multiple IBS mechanisms including increased attention, arousal, and emotion [13], [14]. Several brain imaging studies also observed greater activation of the dorsal pons and midbrain region in IBS patients to rectal distention [15]. In the dorsal brainstem, down-regulation is inhibited in IBS patients during cued expectation [16]. During anticipated conditions, down-regulation is maximal within the dorsal pons after habituation [16]. From this evidence, it appears that IBS patients show different brainstem activities.

IBS patients also show altered central nervous system (CNS) responses to stimuli unrelated to the GI tract [17], [18]. Berman et al. [18] reported a pre-attentive disorder of non-visceral sensory gating as measured by event related potential (ERP). IBS patients show hypersensitivity to various stimuli (such as an exaggerated startle response) and deficits in the ability to habituate to adverse information [17], [18]. Hypersensitivity and deficits to control experimental stimuli may be the key feature of IBS and play an important role in central pain amplification [11]. Therefore, IBS patients have not only visceral hypersensitivity but also hypersensitivity to stimuli unrelated to the GI tract.

The sensitivity of children's brainstems can be examined using the brainstem auditory evoked potential (BAEP) [19], [20]. The amplitude and latency of several electroencephalogram (EEG) waveforms recorded from scalp electrodes in response to specific sensory events are physiological measures of CNS [19], [20]. In particular, the latency of the BAEP wave is an indicator of sensory processing and estimates CNS responsiveness [19], [20]. Although several previous studies evaluated central sensitivity using ERP [18], none have investigated BAEP in children with GI symptoms. Moreover, childhood is an important period for neurodevelopment and is characterized by increased vulnerability to stressors [21], [22]. IBS/RAP children experience more stressful life events in the year before the onset of their symptoms [22], [23], and stressful life events may be associated with abdominal pain in IBS/RAP children [5], [24].

Levy et al. [25] indicated that environmental factors have an equal or greater influence on the development of IBS than genetic factors. Parental overprotection [26] or an unusually high degree of parental anxiety has been shown to have an effect on children's health [27], [28]. Previous studies revealed that patients with psychiatric disorders are apt to receive low parental care or excessive over-protection [28]–[30]. Therefore, it is suspected that alterations of neural pathways along the brain-gut axis could lay the physiological foundations for the integration of life experiences such as sustained parental bonding. However, it is not clear how GI symptoms in children and parental bonding interact with each other. Moreover, how BAEP response in children is influenced by parental bonding style has not been investigated.

In the present study, we investigated the pathogenesis and pathophysiology with neurophysiological features of children at seven. We regarded the age 7 as important period to construct the neurological foundation and the valid period because this age is known to be the pediatric onset of IBS [6]. Moreover, age 7 was used as a reliable age to complete the neurobehavioral testing in the previous study [20]. The aim of this study was to explore the pathogenesis and pathophysiology of FGID in childhood. We tested the primary hypothesis that children with GI symptoms like FGID show exaggerated BAEP responses. We also tested the secondary hypothesis that children with GI symptoms receive more inadequate parental bonding from their parents and the tertiary hypothesis that BAEP response in children is influenced by parental bonding style.

## Materials and Methods

### Subjects

A total of 141 mother-child pairs (73 male children and 68 female children) participated in this study. The healthy participants were recruited without desease, serious mental retardation and mental illness. All children were just 84 months old at the experimental time point and had no audiometric or neurological complaints. Subjects were screened using a medical checklist to exclude current epilepsy or psychoactive medication treatment. No children had inflammatory or other structural diseases as assessed by medical interview. Verbal and written informed consent was obtained from the caretakers of all subjects. This study is part of the Tohoku study of child development but tested hypotheses were completely different from the published study [31]. The present study was approved by Tohoku University Ethics Committee and is performed in accordance with the ethical standards laid down in the Declaration of Helsinki.

### Brainstem auditory evoked potential (BAEP) recordings

The entire experimental session lasted about 2 h. After familiarization with the testing equipment and subsequent equipment fitting, the subjects sat on a chair in a resting state with eyes open in a sound-attenuated air-conditioned and dimly lit room during electroencephalogram (EEG) recording, which has been reported previously [32]. In brief, according to the international 10–20 system, original EEG signals were recorded from scalp Ag/AgCl electrodes and separate ear electrodes A1 and A2. Impedance of electrodes/skin was kept below 5 kΩ.

Segments containing eye movements, blinks, and muscle activity were excluded from analysis. The subjects were instructed to minimize eye blinks and refrain from making movements during the experimental session. On demand, some parents sat beside their children in the testing room during the experimental session. Necessary announcements were given via intercom. The subjects were monitored outside the testing room by a camera system.

The experimental session consisted of two blocks. The first block started with a 75 dB click noise in the right ear and 45 dB white noise masking the opposite ear via headphones; these noises were exchanged from side to side. Stimuli of 75 dB were presented at 0.1 ms duration with a frequency of 20 Hz and an interval of 50 ms. The second block started with a 90 dB click noise in the left ear using the same procedure. Two blocks of 1,000 repetitions were recorded for about 100 s. Data were analyzed using a computer program (Signal Processor 7T-18; NEC Sanei Instruments, Ltd, Tokyo, Japan) and software (EPLYZER, BIMUSTAS; Kissei Comtec, Nagano, Japan) with a 100 ms recording window starting 10 ms after the stimulus onset. Responses were averaged from 2,000 stimuli. All BAEP data were assessed through computerized procedures. Two independent researchers identified BAEP waveforms and measured the latency. Peak latencies were measured in relation to the stimulus [33].

### GI Symptoms and Parental Bonding

The subjects' mothers were administered the Children's Somatization Inventory (CSI) and the Parental Bonding Instrument (PBI) before commencement of the BAEP.

### Children's Somatization Inventory

The Children's Somatization Inventory (CSI) [34], [35] is a self-report questionnaire comprising 35 items and requiring individuals to report the extent to which they experienced each symptom in the previous two weeks: 0 = not at all, 1 = a little, 2 = somewhat, 3 = a lot, 4 = a whole lot. The total CSI score (maximum 140) is the sum of all items reflecting both the range and intensity of experienced symptoms. The CSI has previously been shown to have adequate good internal reliability with coefficient alphas in excess of 0.90. There are four factors in this inventory: pseudoneurological symptoms, cardiovascular symptoms, gastrointestinal symptoms, and pain weakness symptoms.

There are seven items related to GI symptoms like FGID in the CSI questionnaire including abdominal pain, constipation, diarrhea, and abdominal bloating. Using the questionnaire, the children were divided into two groups. Those with a GI score of one or more were classified as the “GI group” and the other children with no GI score were classified as “controls”.

### Parental Bonding Instrument

The Parental Bonding Instrument (PBI) is a well-validated inventory [36], [37] that has also been widely used in medical studies [26], [30]. It is a self-report questionnaire with 25 items that measures parental styles recalled by the responders from the first 16 years of their childhood. The PBI is scored separately for fathers and mothers to subjectively evaluate the relationship between children and each of their parents. Responders were asked to score their attitudes or behaviors using four-point Likert scales (very much like, moderately like, moderately unlike, very unlike). The PBI consists of two factors: the over-protection factor (13 items, a maximum score of 39 and cut-off score 13.5 for mother and 12.5 for father) and the care factor (12 items, a maximum of 36 and cut-off score 27 for mother and 24 for father). Mothers of all subjects completed the PBI both for themselves and the children's fathers.

### Statistical Analyses

All mothers (n = 141) filled out the CSI, but two did not complete the PBI values. Some BAEP values from the right (n = 9) or left side (n = 6) were incomplete because children dropped out during the experimental session because of anxiety or the inability to sit still for a long time.

The Statistical Package for Social Sciences (SPSS), version 12.0 for Windows, was used in all analysis. Results are expressed as the median [interquartile ranges]. Comparisons of differences between two groups were performed by Mann-Whitney U-test. Correlation coefficients were calculated with Spearman's Rho. Multiple regression analysis was performed to determine the relationship between GI scores and BAEP parameters, parental care, parental overprotection, and CSI scores, with the exception of GI scores. Statistical significance was judged by a *p*-value less than 0.05.

## Results

### Children with GI symptoms screened by CSI

There were 66 (42%) children (37 males and 29 females) without GI symptoms (controls). Seventy-five (58%) children (36 males and 39 females) had one or more GI symptoms and were classified as the GI group. GI scores of the GI group ranged from 1 to 21 with a mean of 2.3 and a SD of 2.7.

### Comparison of BAEP peak latency between controls and GI group

**Figure 1:**
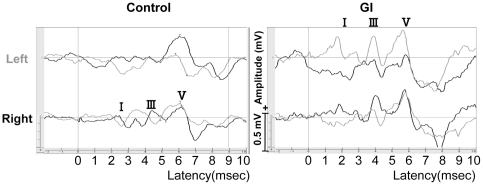
Actual wave forms of the brainstem auditory evoked potential (BAEP) in controls and FGID children. Upper lines are BAEPs with left ear stimulation, lower lines are BAEPs with right ear stimulation. Gray lines indicate recordings of left side, black lines show those of right side. Note remarkable and positive I, III, and V waves in ipsilateral recordings of stimulated sides, and vague wave forms in contralateral recordings of opposite sides. Note shorter latency of III wave in GI child compared with control.

We obtained remarkable and positive I, III, and V waves in ipsilateral recordings of the stimulated sides and vague wave forms in contralateral recordings of the opposite sides (**Figure 1**). In the ipsilateral recordings, the original III wave form of the GI group showed a shorter latency than that of controls.

**Figure 2:**
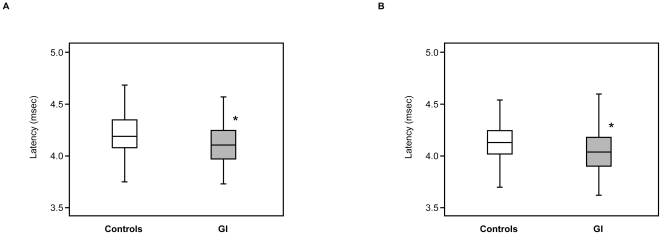
Peak latency of BAEP III wave in all children. **A**: right ear, □open box: controls (n = 63), ▪shaded box: GI group (n = 69). **B**: left ear, □open box: controls (n = 62), ▪shaded box: GI group (n = 73). Values (msec) are given as median [interquartile range] (minimum-maximum). Asterisk (*) indicates significant difference versus controls at *p* = 0.032 in the right and *p* = 0.018 in the left.

In the right side, the latency of the III wave in the GI group (4.10 [3.95–4.24] ms) had a significantly shorter peak latency than that in controls (4.18 [4.06–4.34] ms, *p* = 0.032) (**Figure 2A**). In the left side, the latency of the III wave in the GI group (4.04 [3.90–4.18] ms) had a significantly shorter peak latency than that in controls (4.13 [4.02–4.24] ms, *p* = 0.018) (**Figure 2B**). By contrast, there was no significant difference in I and V waves between the two groups (**Table 1**).

**Table 1:** Latency of I and V Waves of the Brainstem Auditory Evoked Potential.

Latency (msec)	controls	GI	p-value
All subjects (number)	63 (right)/62 (left)	69 (right)/73 (left)	
I wave (right)	1.96 (1.83–2.10)	1.94 (1.84–2.04)	0.629
I wave (left)	1.85 (1.72–2.04)	1.82 (1.74–2.00)	0.563
V wave (right)	5.88 (5.79–6.06)	5.88 (5.74–6.02)	0.460
V wave (left)	5.84 (5.74–6.00)	5.82 (5.66–5.96)	0.405
Female (number)	27 (right)/27 (left)	35 (right)/37 (left)	
I wave (right)	1.96 (1.84–2.07)	1.90 (1.81–2.02)	0.303
I wave (left)	1.86 (1.79–2.12)	1.78 (1.72–1.90)	0.133
V wave (right)	5.82 (5.76–5.87)	5.84 (5.75–5.92)	0.594
V wave (left)	5.76 (5.67–5.88)	5.76 (5.54–5.92)	0.629
Male (number)	36 (right)/35 (left)	34 (right)/36 (left)	
I wave (right)	1.96 (1.83–2.22)	1.98 (1.88–2.14)	0.659
I wave (left)	1.84 (1.71–2.02)	1.84 (1.76–2.02)	0.519
V wave (right)	6.00 (5.87–6.11)	5.95 (5.74–6.12)	0.394
V wave (left)	5.90 (5.80–6.03)	5.87 (5.72–6.02)	0.743

Data are expressed with median [interquartile range].

### BAEP peak latency in female and male GI groups

**Figure 3:**
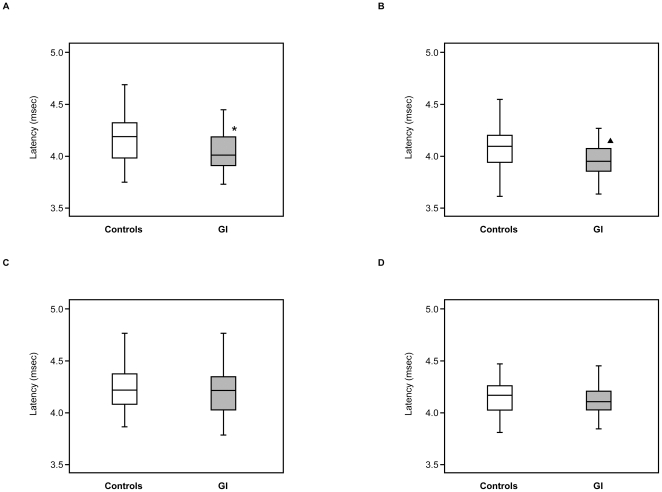
Peak latency of BAEP III wave in females and males. **A**: female, right ear, □open box: controls (n = 27), ▪shaded box: GI group (n = 35), **B**: female, left ear, □open box: controls (n = 27), ▪shaded box: GI group (n = 37), **C**: male, right ear, □open box: controls (n = 36), ▪shaded box: GI group (n = 34), **D**: male, left ear, □open box: controls (n = 35), ▪shaded box: GI group (n = 36). Values (ms) are given as median [interquartile range] (minimum-maximum). Asterisk (*) indicates significant difference versus controls at *p* = 0.034. Solid triangle (^▴^) indicates tendentially but not significantly different from controls at *p* = 0.059.

The female GI group had a significantly shorter peak latency of the III wave in the right side (4.00 [3.90–4.18] ms) than controls (4.18 [3.97–4.31] ms, *p* = 0.034) (**Figure 3A**). There was a tendentially but not significantly shorter peak latency of the III wave in the left side of the female GI group (3.94 [3.84–4.06] ms) than that of controls (4.08 [3.93–4.19] ms, *p* = 0.059) (**Figure 3B**). By contrast, the male GI group showed the same peak latency of the III wave in the right side (4.21 [4.02–4.34] ms) and left side (4.10 [4.02–4.20] ms) compared with the controls in the right side (4.21 [4.08–4.37] ms, *p* = 0.668) and left side (4.16 [4.02–4.25] ms, *p* = 0.279) (**Figure 3C, 3D**). There was no significant difference in the other components between the two female groups or two male groups (**Table 1**).

### Correlation between GI symptoms and BAEP latency of III wave

The number of GI symptoms slightly but significantly correlated with the peak latency of the III wave on the left side (rho = −0.19, *p* = 0.028). GI scores also showed a significant correlation with the peak latency of the III wave on the left side (rho = −0.19, *p* = 0.025).

### BAEP peak latency and somatization

We divided children into two groups according to the median (5) total CSI score. There was no significant difference in the peak latency in any component between children with high somatization and those with low somatization (data not shown).

### PBI and GI symptoms

**Figure 4:**
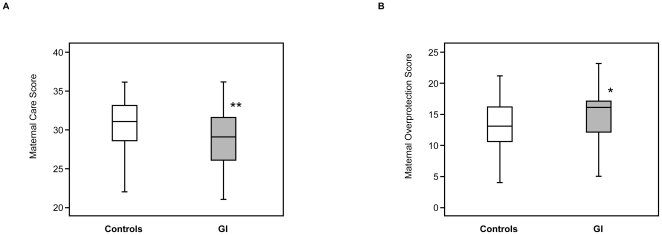
The care score and over-protection score of Parental Bonding Instrument in controls and FGID children. **A**: maternal care, **B**: maternal overprotection, □open box: controls (n = 64), ▪shaded box: GI group (n = 75). Values (ms) are given as median [interquartile range] (minimum-maximum). Double asterisk (**) indicates significant difference versus controls at *p* = 0.010 (A). Asterisk (*) indicates significant difference versus controls at *p* = 0.024 (B).

The maternal care PBI scores in the GI group (29 [26]–[33]) were significantly lower than those of controls (31 [28.5–33], *p* = 0.010) (**Figure 4A**). In addition, the PBI maternal care score showed a significantly negative correlation with GI scores (rho = −0.22, *p* = 0.010). The maternal over-protection PBI scores in the GI group (16 [12]–[17]) were significantly higher than those of controls (13 [10.5–16], *p* = 0.024) (**Figure 4B**) and showed a significantly positive correlation with GI scores (rho = 0.19, *p* = 0.023). There was no significant difference in paternal care between GI group and controls.

### Correlation between PBI and BAEP latency of III wave

There was no significant correlation between PBI scores and BAEP latency of III wave (data not shown).

### Multivariate analysis between GI symptoms and CSI/PBI/BAEP

Multiple regression analysis revealed that GI scores were significantly predicted by CSI (except GI) score (β = 0.833, *p* = 0.0001), maternal care (β = −0.160, *p* = 0.014) and paternal care (β = 0.174, *p* = 0.007) and tendentially but not significantly predicted by latency of III wave in the left side (β = −0.163, *p* = 0.058) (R^2^ = 0.699, *p* = 0.0001)(**Table 2**). Moreover, multiple regression analysis in female revealed that GI scores were significantly predicted by CSI (except GI) score (β = 0.550, *p* = 0.0001), maternal care (β = −0.384, *p* = 0.001), paternal care (β = 0.345, *p* = 0.003) and latency of III wave in the right side (β = −0.257, *p* = 0.015) (R^2^ = 0.495, *p* = 0.0001). By contrast, multiple regression analysis in male revealed that GI scores were only predicted by CSI (except GI) score (β = 0.873, *p* = 0.0001) (R^2^ = 0.775, *p* = 0.0001).

**Table 2:** Multiple regression analysis for total sample.

independent variable	standardized β	*p*-value
CSl (except GI) score	0.833	0.0001
paternal care	0.174	0.007
maternal care	−0.160	0.014
latency of III wave (left)	−0.163	0.058
latency of V wave (left)	0.083	0.411
maternal overptotection	−0.054	0.429
latency of I wave (right)	−0.032	0.702
latency of V wave (right)	0.033	0.741
paternal overprotection	0.021	0.754
latency of III wave (right)	−0.022	0.826
latency of I wave (left)	−0.010	0.896

GI symptoms as dependent variable and CSI (except GI) score, parental care, over-protection, and latencies of BAEP as independent variables. R^2^ = 0.699, *p* = 0.0001.

## Discussion

This is the first study to demonstrate an exaggerated response in the brainstem to auditory stimuli in children with GI symptoms. The shorter latency of the BAEP III wave in children with GI symptoms at supports our main hypothesis. By contrast, regarding somatization, there is no significant difference in peak latency of all components compared with controls. Therefore, differences in the peak latency of the III wave may be related to IBS-like symptoms. Peaks of the I, III, and V waves are thought to reflect volume-conducted electrical activity from the acoustic nerve, pons (superior olivary nucleus), and midbrain (inferior colliculi) [33]. The latency of the peak III component of BAEP reflects the brainstem response [33] and is known to increase with age [38]. In previous studies [20], the latency of the BAEP III wave in seven-year-old Japanese children positively correlated with their hair mercury concentration, reflecting neurodevelopmental toxicity caused by methylmercury exposure. The latency of the III wave was previously shown to be negatively correlated with the number of cigarettes smoked during the three months prior to pregnancy [39]. Wang et al. [40] also revealed a positive relationship between BAEP and the clinical state of cognition in Alzheimer's disease. Given this background, our findings may reflect a developmental deficit of the brainstem as well as an exaggerated response to auditory stimuli but not minor brain damage.

The BAEP findings in the present study are in the same direction with the earlier studies. Berman et al. [18] revealed that IBS patients show an enhanced large P_1_ of ERP due in part to the activity of the pontine region associated with the cholinergic ascending arousal system. The brainstem locus coeruleus is the primary source of ascending noradrenergic projections that mediate arousal and form a positive feedback loop with corticotropin-releasing hormone-containing neurons in the amygdala [41], [42]. There is no clear explanation of female predominant shorter latency of BAEP III wave. However, the locus coeruleus is known to be larger in females than in males [43]. Moreover, brain imaging studies showed that female IBS patients were unable to downwardly regulate the homeostatic afferent processing that occurs during normal anticipation of visceral pain [16], and demonstrate increased activation in the dorsal brainstem and anterior cingulate cortex [16]. The biological substrate functioning as top-down or bottom-up modulation to external stimuli in IBS/RAP children may contribute to enhanced brainstem responsiveness, while the shorter latency of the BAEP III wave clearly seen in female children with GI symptoms may be due to altered perceptual responses to afferent signals. Thus, our main hypothesis was partially supported by results from female children with GI symptoms. Further study is needed to reject the shorter latency of BAEP III wave in male children with GI symptoms.

Our CSI and PBI results support the secondary hypothesis that this study set out to test, namely that children with GI symptoms receive more inadequate parental bonding, such as imbalance of parental bonding, reduced maternal care, excessive paternal care, or overprotection from their parents. The tertiary hypothesis that PBI scores relate to BAEP was not supported. Therefore, inadequate parental bonding and BAEP are independent factors that affect GI symptoms. Adverse childhood experiences are proven risk factors for the development of many diseases [26], [29], [30] and an association between adverse parenting and abnormal cortisol levels has been reported in previous studies [44], [45]. Alterations in the hypothalamic-pituitary-adrenal (HPA) axis and sympathetic nervous system (SNS) have been reported in several stress-related disorders [46], [47]. Many studies revealed that maladaptive HPA responses and SNS function relate to FGID such as IBS [48]–[50]. Exposure to adverse environments such as inadequate parenting during childhood may lead to serious life-long effects due to impaired brain development and dysregulation of the brain-gut axis [1], [51]. Moreover, chronic sustained stress, particularly as a primary life event, has been demonstrated to be an important factor in both FGID onset and modulation [49], [52]. The mother's parental style during childhood may be associated with dysregulation of emotional inhibition [53], [54] and the onset of GI symptoms [55], [56].

In this study, maternal overprotection beyond normal range and reduced maternal care even within normal range among inadequate parental behaviors are likely to play some roles in childhood GI symptoms. Clinical and epidemiological studies suggest that adverse parenting characterized by low care is a significant risk factor in the development of depressive disorder [30]. Some studies reported that adverse experiences in childhood are mediated by other long-standing vulnerability factors [30], [45]. For example, Walker et al. [23] reported that child patients with abdominal pain have higher levels of anxiety and depression than those without pain, while Levy et al. [25] clarified the mechanism of frequent GI complaints in children based on solicitous responses to illness from their mothers. Childhood and adolescence are important periods for neurodevelopment and maturation of brain regions [57]. While our study researches a similar area to these previous investigations, it builds on the earlier studies by depicting specific parenting styles that increase the risk of developing FGID.

Linear regression analysis revealed that paternal care and maternal care are the predictable factors associated with GI symptoms. In this study, it is not clear why the score of maternal care was negatively related to GI symptoms, but paternal care was positively related to GI score. It is possible that the responders' recall bias may affect these opposite result. Janssens et al [26]. reported that parental bonding may play a role in the development of functional somatic symptoms, and several studies indicated that IBS patients have a hypervigilance for symptom-relevant sensations [58], [59]. The number of GI symptoms is likely to be associated with hypersensitivity. IBS patients show a decreased ability to refocus attention away from bothersome stimuli under chronic heightened autonomic arousal [16]. Hypersensitivity may therefore be associated with attentional and affective modulation of perception in IBS [51]. Neuroplastic and structural alterations have been observed in the CNS in response to sustained severe life stress [46], [47], including presynaptic and postsynaptic changes in ascending monoaminergic arousal systems and the HPA axis [46], [47], which are likely to be related to functional GI symptoms. The relationship between bonding and GI symptoms may go in both directions: either the way of parental bonding changes because the child has GI symptoms (and as a reaction the mother is more over-protective etc.) or the child develops GI problems because of bad parenting. Further research to testify the influence of generation, culture, and gender of caregiver on parental bonding and GI symptoms of children will be available.

This study has some limitations. First, PBI was retrospectively used for measuring the behavior of parents and as such we could not avoid recall bias. However, PBI reliability has been confirmed in previous studies [26], [29], [30], so this limitation is in line with the earlier works. Second, we cannot rule out the possibility that the fact itself that their children had GI symptoms might influence the mothers' replies to PBI. In addition, the mothers' replies to PBI about their husband might be influenced by their relationship. Third, it is difficult to use a questionnaire to measure emotion in seven-year-old children, and therefore this state could not be assessed. Last, we assessed children with IBS-like symptoms using reports from their mothers rather than the results of colonoscopy, radiological examination, or histological biopsy. However, colonic cancer and inflammatory bowel disease among this population is negligible [60]. This study design was done from an ethical point of view to prevent risk to the children.

In conclusion, our study suggests that children with GI symptoms show exaggerated BAEP responses and that they receive more inadequate parental bonding from their parents. Further studies are warranted to explore the pathogenesis and pathophysiology of FGID in childhood together with neurodevelopment.
